# Alcohol Recognition by Flexible, Transparent and Highly Sensitive Graphene-Based Thin-Film Sensors

**DOI:** 10.1038/s41598-017-04636-2

**Published:** 2017-06-28

**Authors:** Xuezhu Xu, Jian Zhou, Yangyang Xin, Gilles Lubineau, Qian Ma, Long Jiang

**Affiliations:** 1 King Abdullah University of Science and Technology (KAUST), Physical Science and Engineering Division, COHMAS Laboratory, Thuwal, 23955-6900 Saudi Arabia; 2 North Dakota State University, Department of Mechanical Engineering, Fargo, ND 58102 United States; 3 College of Textile and Clothing, Yancheng Institute of Industry Technology, Yancheng, 224005 P. R. China

## Abstract

Chemical sensors detect a variety of chemicals across numerous fields, such as automobile, aerospace, safety, indoor air quality, environmental control, food, industrial production and medicine. We successfully assemble an alcohol-sensing device comprising a thin-film sensor made of graphene nanosheets (GNs) and bacterial cellulose nanofibers (BCNs). We show that the GN/BCN sensor has a high selectivity to ethanol by distinguishing liquid–phase or vapor–phase ethanol (C_2_H_6_O) from water (H_2_O) intelligently with accurate transformation into electrical signals in devices. The BCN component of the film amplifies the ethanol sensitivity of the film, whereby the GN/BCN sensor has 12400% sensitivity for vapor-phase ethanol compared to the pure GN sensor, which has only 21% sensitivity. Finally, GN/BCN sensors demonstrate fast response/recovery times and a wide range of alcohol detection (10–100%). The superior sensing ability of GN/BCN compared to GNs alone is due to the improved wettability of BCNs and the ionization of liquids. We prove a facile, green, low-cost route for the assembly of ethanol-sensing devices with potential for vast application.

## Introduction

Sensor devices aim to detect changes in the physical environment (i.e., light/heat/motion/moisture/pressure/chemicals) and to convert them into readable signals. The demand for sensor devices has been growing in several fields, including automobile, aerospace, safety, indoor air quality, environmental control, food, industrial production and medicine. Incorporation of graphene into sensors in flexible electronic devices is central^1, 2^ because it offers high flexibility, high tensile modulus (1 TPa), high carrier mobility (200,000 cm^2^·V^−1^·s^−1^), large specific surface area (2,630 m^2^·g^−1^)^3^, good thermal conductivity (~5000 W·m^−1^·K^−1^) and excellent optical transmittance (~93%)^4^. Sensing devices have been developed to detect volatile and gaseous molecules^5^, such as nitric oxide^6^, acetone^7^ and ethanol^8^. Compared with traditional approaches for fabricating ethanol-sensing devices using metal oxides (i.e. Al_2_O_3_, V_2_O_5_)^9^, graphene offers a lot of advantageous features when used in sensors. For example, its pliability makes graphene-based sensors flexible, a property that is relatively difficult to achieve in an inorganic component of a sensing device. Graphene is also highly conductive, so it does not require additional doping with conductive filler, and its high specific surface area makes it sensitive to the environment. For these reasons, we used graphene nanosheets (GNs), typically comprising fewer than three sheets, along with a relatively new building block, bacterial cellulose nanofibers (BCNs), to assemble an alcohol-sensing device^10^.

Cellulosic nanofibers are one of three emerging nanomaterial fibers (cellulosic, carbon and inorganic nanofibers) that can be used for device assembly^11^. BCNs are extracted from a hydrogel produced by the bacteria *gluconacetobacter xylinus*, which can be harvested when the bacteria are grown on culture mediums. BCNs comprise polysaccharides (C_6_H_10_O_5_)_n_ made of many thousands of β(1 → 4) linked D-glucose units. BCNs are highly crystalline (up to 90%) and have fibers that are ultrafine (2–50 nm) in diameter and that have rich surface chemistry^12^, high flexibility, high tensile strength (>1 GPa) and high tensile moduli (78–114 GPa)^13–16^. Their high intrinsic water absorbance makes them suitable as raw materials for preparing foams with high porosity when water is removed^17–22^. Furthermore, BCNs can be used as templates to grow various inorganic particles, such as silver nanowires (Ag) and ferromagnetic materials (CoFe_2_O_4_)^23, 24^. Based on these characteristics, cellulose nanofibers have been adopted in some applications, including solar cells^25^, electrodes^26^, transistors^20^ and light-emitting diodes^27, 28^. We are interested in the rich surface chemistry and high absorbance of BCNs and predict that BCNs will have a synergistic relationship with GNs in sensor devices.

In this study, BCNs are designated as the matrix to host GNs to achieve the desired typical tendency of a microstructure like that of BCNs’ to “wrap” around GNs. The GN/BCN composite forms a thin film, which is capable of serving as a sensing material. We predict that the composite will produce a superior electrical signal in response to ethanol and water in both liquid and vapor phases.

## Results and Discussion

### Assembly process from raw material to sensor device

**Figure 1:**
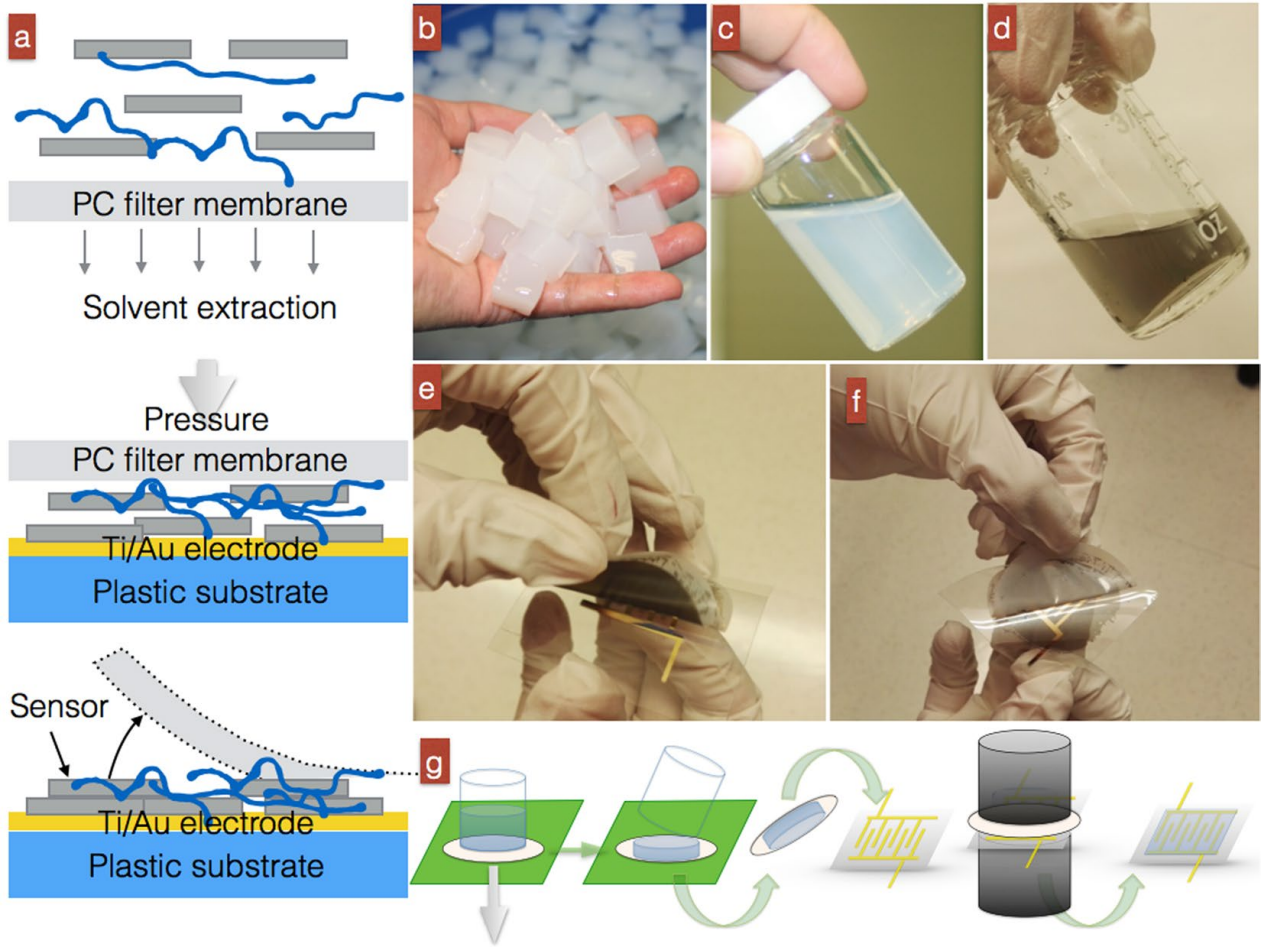
(**a**) Preparation of a GN/BCN sensor by vacuum-filtration and lamination technique. (**b**) Pristine BC hydrogels at 0.5 wt.%. (**c**) 1 wt.% aqueous BCN suspension after a TEMPO-mediated extraction process. (**d**) GN/BCN colloidal mixture with 40 wt.% GN content. (**e**) Lamination of a wet substrate-supported GN/BCN device at 40 wt.% GNs on a piece of plastic substrate sputtered with interdigitated Ti/Au electrodes. (**f**) Process of peeling the filter substrate from the dried GN/BCN sensor after vacuum compressing/drying at 60 °C for 4 h. (**g**) Complete process of device assembly.

We assembled the GN/BCN devices via vacuum filtration followed by lamination process (Fig. 1a)^29–32^. Figure 1b shows BC hydrogel pellicles with a solid weight content of 0.5 wt.%. The gel contains many ultrafine nanofibers with water filling as high as 99.5 wt.% in the nanofiber network. The BCNs were obtained by grinding these BC pellicles in a kitchen blender (for 5 min) followed by mild stirring assisted with 2,2,6,6-tetramethylpiperidine-1-oxyl (TEMPO) radical mediation. TEMPO mediation is a treatment commonly used in woody industries to destroy fiber-fiber chemical bonding. Figure 1c shows the aqueous BCN suspension at a concentration of 0.1 wt.%, obtained following purification. Although the extraction of BCNs is similar to that for plant-based cellulose nanofibers^33^, the difference is that BCNs are based on bacterial cellulose pellicle, which can be grown on a large scale at very low cost. Their extraction is therefore facile and energy efficient. They are also desirable because they are considered to be a “green” supply with near-zero energy consumption during the extraction process compared to the energy consumption of plant-based nanofibers, which is often as high as 22, 2.8 and 0.5–2.3 kWh/kg for homogenization, microfluidization and chemical/enzyme treatment^34, 35^. After extraction, the aqueous BCN suspension was mixed with different concentrations of GN powder to form GN/BCN mixtures (Fig. 1d). The mixtures were loaded into vacuum-filtration equipment to form wet thin films with residual solvent (Fig. 1g). The film, supported by a filter membrane, was placed face down on top of a plastic substrate that had been deposited with interdigitated Titanium/gold (Ti/Au) (10, 100 nm thick, respectively) electrodes (Fig. 1e). These electrodes collect the electrical signals of the GN/BCN sensor. Ti was used to increase the adhesion between the Au layer and the plastic substrate. After compressing/drying, the filter membrane was peeled away from the surface of the GN/BCN film leaving behind a dry GN/GCN thin film deposited on the substrate (Fig. 1f).

### Structure of the GN/BCN network

**Figure 2:**
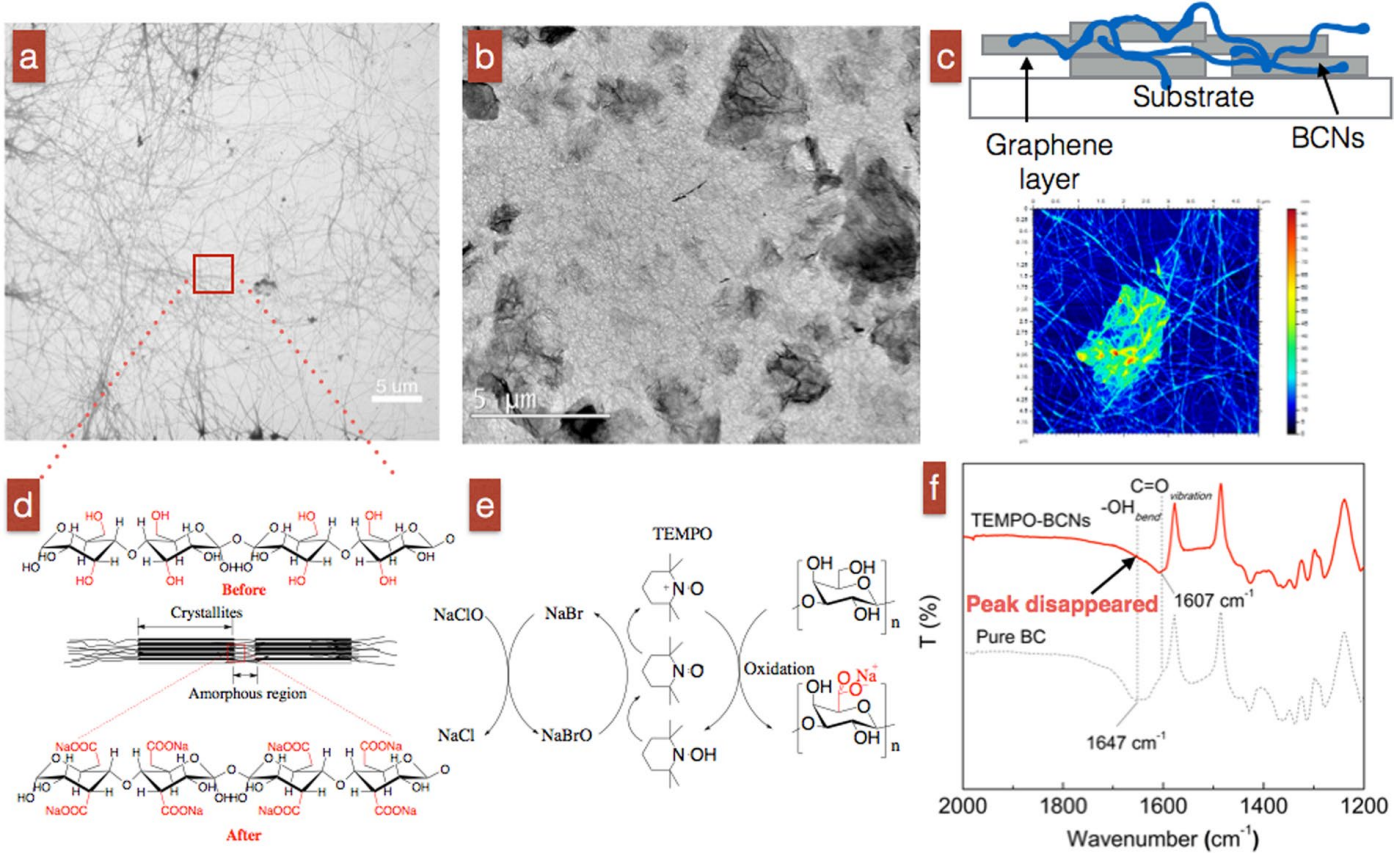
(**a**) TEM image of TEMPO-mediated BCNs derived from BC gels. (**b**) TEM image of a GN/BCN films with a GN 40 wt.%. (**c**) Scheme and atomic force microscopy image of the structure of a GN/BCN sensor. (**d**) Molecular structure of BCNs before and after TEMPO modification. (**e**) Chemical reaction on BCNs caused by TEMPO-modification^43^. (**f**) FT-IR spectra of TEMPO-modified BCNs and pristine BCNs.

Figure 2a shows a TEM image of TEMPO-mediated BCNs with an average diameter (D) of 9.9 nm. We measured fiber length (L, within visible area) at a minimum of 5.5 μm (fibers were very curved and knitted), and therefore, their aspect ratio (L/D) was estimated to be in the order of 5000. These fibers are relatively long, compared to either ~1 μm long wood-derived cellulose nanofibers, or ~hundreds of nm long cellulose nanocrystals^10^. Long fibers with a high aspect ratio like those of TEMPO-mediated BCNs are believed to have good mechanical properties (i.e., flexibility in devices)^10^. Figure 2b,c show graphene sheets randomly distributed within the fibrous BCN network. BCNs contain abundant hydroxyl groups (C-OH) on their surfaces due to their intrinsic cellulosic origin (Fig. 2d). These groups change into carboxyl groups (O=C-O-Na) after TEMPO modification (Fig. 2e). The successful modification made to the BCN fibers by TEMPO oxidization was evidenced by the disappearance of the –OH peak at 1647 cm^−1^ and the increase in the C=O peak at 1607 cm^−1^ (Fig. 2e). This section proves that TEMPO-treated BCNs were successfully obtained; homogenous mixing of GNs with BCNs facilitates formation of a GN/BCN composite thin film.

### Electrical and optical properties of GN/BCN films

**Figure 3:**
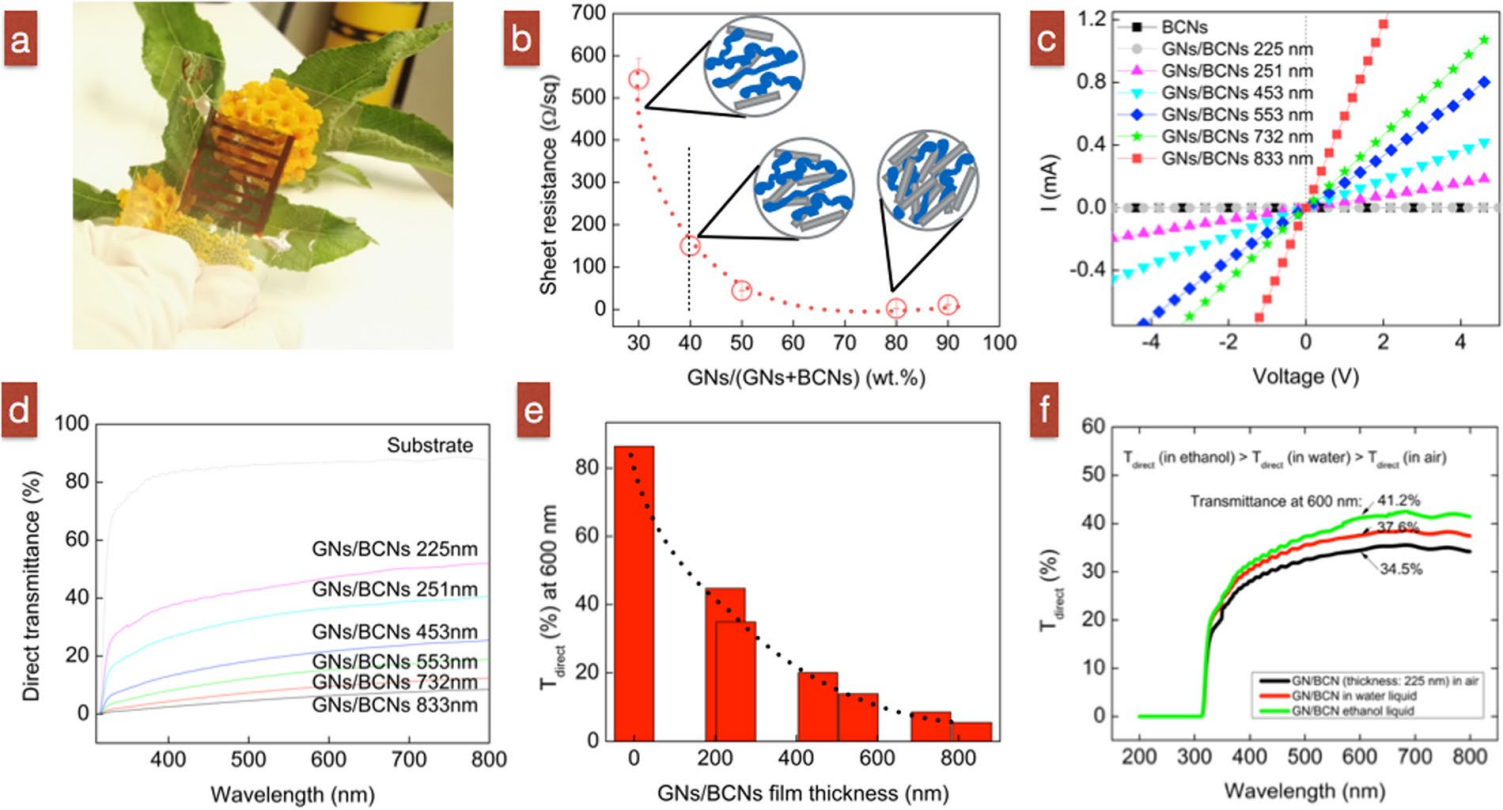
(**a**) A 225-nm-thick GN/BCN sensor. (**b**) Dependence of sheet resistance of GN/BCN thin films on the concentration of GNs in the sample. (**c**) I-V characteristics of GN/BCN thin films of varied thicknesses. (**d**,**e**) Direct transmittance at 600 nm for GN/BCN of different thicknesses. (**f**) Transparency of a piece of GN/BCN thin film (thickness = 225 nm) exposed to air, ethanol or water.

Figure 3 illustrates our studies of the electrical and optical properties of GN/BCN thin films. Figure 3a shows a 225-nm-thick piece of the semi-transparent GN/BCN film with 40 wt.% GNs concentration (ϕ). This value was determined as the critical threshold, η_c_, which was obtained from the relationship of electrical conductivity and GN concentration for a GN/BCN film (Fig. 3b). At this point, the GNs form a percolated conductive network with conductive particles “just-connected” rather than “under-connected” or “overlapped” (see Fig. 3b illustration). In other words, we are very close to the electrical percolation point. When the GNs are just connected, the vacancies between GNs absorb molecules from the environment, which can cause a quick upshift or downshift in electrical conductivity. Figure 3c plots the current–voltage (I–V) characteristics of 225–833-nm-thick GN/BCN thin films using 40 wt.% GNs measured under voltages between −5 and +5 V. The linear I–V curves indicate that satisfactory Ohmic contact is achieved between the GN/BCN films and the interdigitated Ti/Au electrode. The reciprocal of the I–V slope represents the resistance, and these data show that thinner GN/BCN films have higher electrical resistance. Figure 3d shows the direct transmittance, T_direct_, against wavelengths by various GN/BCN thin films. T_direct_ is defined as the amount of light that passes through the sample within 2.5° with respect to the total amount of light that passes through the sample. Figure 3e shows that 225-nm-thick GN/BCNs have 50% direct transmittance, which attests to the high transparency of both GNs^4^ and BCNs^28–32, 36, 37^.

We focus on high transparency because we expect that we will integrate our thin-film sensor into or laminate it onto any electronic device (touch screens, flexible displays, printable electronics, solid-state lighting and thin-film photovoltaic) without deteriorating the optical properties when it functions to detect alcohol. We quantified the dependence of transparency (direct transmittance, T_direct_) on the GN/BCN thin-film sensor when it was covered with either ethanol or water. As shown in Fig. 3f, the transparency was 34.5% (transparency varied from 30 to 50% among samples) for a GN/BCN thin film exposed to air. The transparency increased slightly to 37.6% when exposed to water. It then reached its maximum at 41.2%. In general, we ascribed these changes to the smooth surface of the liquid films that decreased the surface roughness. A decreased surface roughness caused a decreased scattering factor, which allowed more light to pass through the GN/BCN film.

### Sensing behavior to liquid-phase and vapor-phase ethanol and/or water

**Figure 4:**
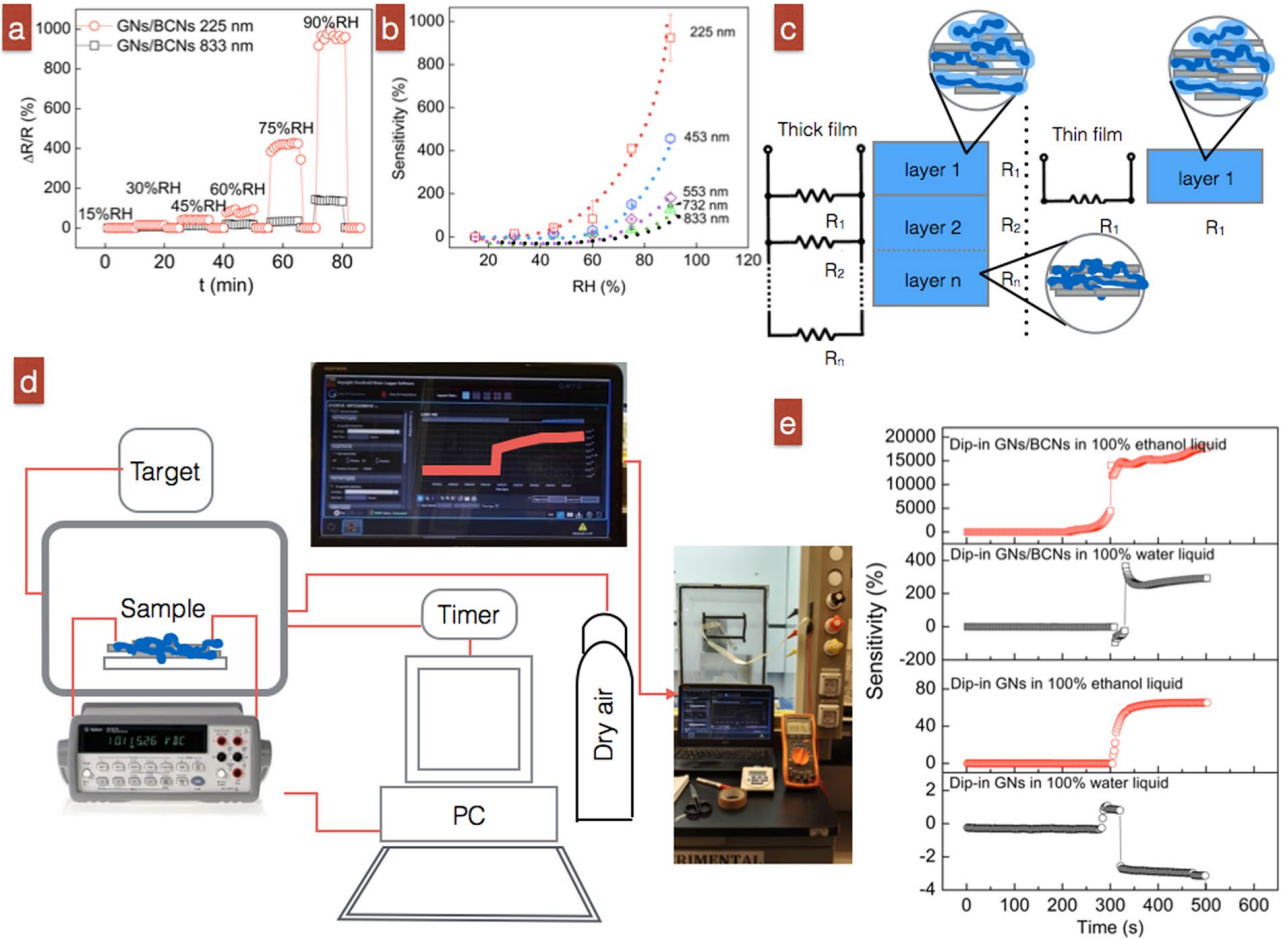
(**a**) Representative resistivity changes of 225-nm-thick and 833-nm-thick GN/BCN sensors at various RH. (**b**) Sensitivity of GN/BCN sensors when exposed to air at different %RH. (**c**) Electrical configuration models in thick and thin GN/BCN films. (**d**) Experimental setup to evaluate the sensing behavior of GN/BCN sensors when exposed to alcohol or water vapors. (**e**) Sensitivity performance of GN/BCN sensors detecting liquid-phase ethanol and water.

When applying GN/BCN film sensors practically, we used sensitivity (∆R/R, %) to evaluate their sensing performance when exposed to target liquids or vapors:

1 $$Sensitivity=\frac{{\rm{\Delta }}R}{R}=\frac{{R}_{intarget}-{R}_{inair}}{{R}_{inair}}\times 100 \% \,,$$

where ΔR is the change in resistance, R is the original resistance, R_in target_ is the real-time resistance as the sensing device is exposed to the target and R_in air_ is the initial resistance for the device in air. First, a relative humidity (RH) test was performed to test the response of GN/BCN films to controlled humidity; this was done using exposure/recovery cycles at 15, 35, 45, 55, 60, 75 and 90% RH at 10-min intervals. The thinnest GN/BCN devices (~225 nm) had the highest sensitivity: 0, 15, 42, 90, 420 and 966% at 15, 35, 45, 60, 75 and 90% RH (Fig. 4a). GN/BCN films of various thicknesses were tested to probe the relationship between sensitivity and film thickness. We observed that for all samples, GN/BCN devices had increasing electrical resistance with increasing RH (Fig. 4b), We explain the higher sensitivity of devices made with thinner than with thicker films by considering the diffusivity in the layered structure as parallel circuits. In a thick film with R_1_, R_2_ and R_n_ resistive layers (Fig. 4c), total resistance (R_total_) is defined as:

2 $$\frac{1}{{R}_{total}}=\frac{1}{{R}_{1}}+\frac{1}{{R}_{1}}+\ldots +\frac{1}{{R}_{n}}$$

For example, if we assume a thick film consists of two layers and a thin film only has one layer, their total resistance becomes:

3 $${R}_{total}=\frac{{R}_{1}{R}_{2}}{{R}_{1}+{R}_{2}}\,(thick\,film)$$

4 $${R}_{total}={R}_{1}\,(thin\,film)$$

Assuming in dry air, the initial values of each layer is R_1_ = R_2_ = 1 Ω, then initial R_total_ is 0.5 Ω for the thick film and 1 Ω for the thin film. When the first layer is exposed to targets (H_2_O, C_2_H_6_O), we assume again that the resistance R_2_ remains the same, R_1_ gets doubled (i.e., R_1_ = 2 Ω). Thus, R_2_ = 1 Ω so R_total_ = 0.7 and 2 Ω for thick and thin films, respectively. Sensitivity (ΔR/R) was calculated to be 17% and 100% for thick and thin GN/BCN films, respectively. This simple model demonstrates that a thin-film GN/BCN sensing device has high sensitivity when exposed to target liquids.

Next, the real-time response of a GN/BCN sensor (225-nm thick) to liquid-phase ethanol and water was tested (Fig. 4d). This required that we record the electrical resistance of the GN/BCN film when exposed to dry air followed by a quick insertion of the film into a container filled with pure liquid ethanol (or water) for 5 s. Once removed, the sensor was left to air dry for 5 min. The GN/BCN device showed different sensitivity in response to ethanol and water (Fig. 4e). For example, GN/BCN films had a sensitivity as high as ~15700% to pure liquid ethanol and a sensitivity of 292% to pure liquid water (small irregularities at time of 300 s were caused by the 5 s immersion in target liquids). In comparison, pure GN sensors had a sensitivity of 65% to pure liquid ethanol and a sensitivity of −14% to pure liquid water.

**Figure 5:**
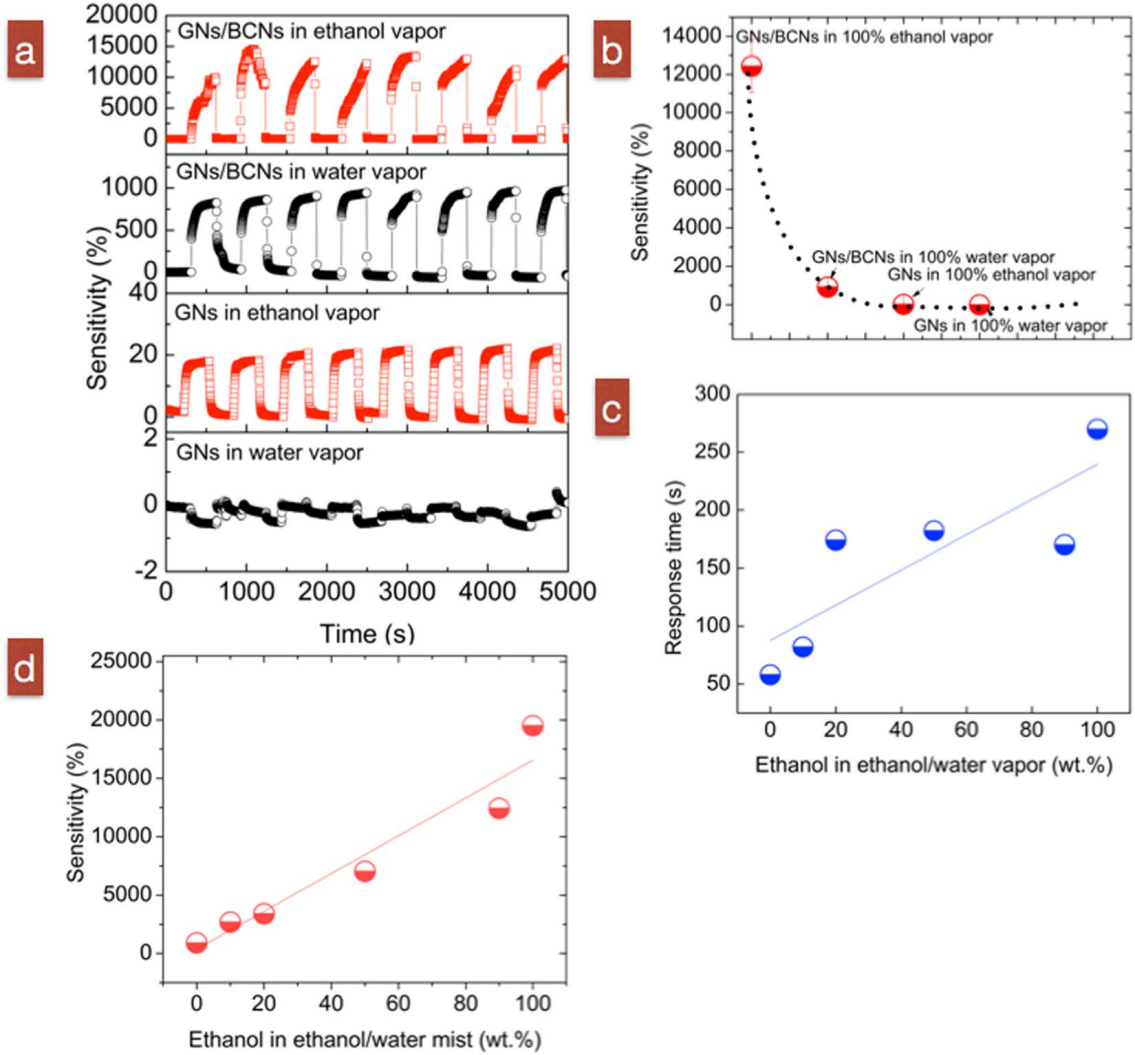
(**a**) Representative sensitivity curves of 225-nm-thick GN/BCN sensors detecting pure ethanol and pure water vapor. GN-based sensor was tested as a control. (**b**) Comparison of the sensitivities of GN/BCN and GN-based sensors. (**c**,**d**) Response time and sensitivity of 225–nm-thick GN/BCN sensors relative to ethanol concentration.

Sensing behaviors of 225-nm-thick GN/BCN devices in response to vapor targets using pure ethanol, pure water and ethanol/water mixtures were further investigated. Figure 5a shows the cyclic sensing test with a 5-min exposure/recovery interval time of GN/BCN sensors. It also illustrates representative cyclic electrical curves of the sensor performed under pure ethanol and in air and compares those with the sensing performance of GN/BCN films with exposure to pure water in the vapor phase. Sensitivity of pure GN sensors to the same target vapors is also presented. Average sensitivities for GNs/BCNs to various vapor targets are plotted in Fig. 5b. The composite GN/BCN device had much higher sensitivity, achieving up to ~12400% sensitivity in response to pure ethanol vapor and 920% sensitivity in response to pure water vapor compared with the pure GN sensor with 21% sensitivity in response to pure ethanol and −1% in response to 100% water. Overall, sensing behavior was similar in response to vapor targets and liquid targets. Thus, although both types of sensors prove to be smart devices, demonstrating intelligence by “telling” us if the target is water or ethanol via an electrical signal, the composite does so with much higher sensitivity than the pure GN sensor.

The GN/BCN sensor exhibits clear response and recovery behavior and acceptable repeatability: response time and recovery time of 108–147 s and ~0 s, respectively (Fig. 5c). We measured the response of the hybrid sensor to ethanol/water vapor mixtures at varied mass ratios. On average, the sensor exhibited a positive relationship with respect to sensitivity to ethanol concentration at 10–90% (Fig. 5d. The fitting equation for sensitivity Y and ethanol concentrations X is represented as Y = 1.52X + 87.72 with a standard deviation (SD) of 29%. These results verify GN/BCN films as suitable smart sensors with low-energy consumption, fast response, high selectivity and rapid recovery characteristics.

### Relationship between sensitivity and target liquids

**Figure 6:**
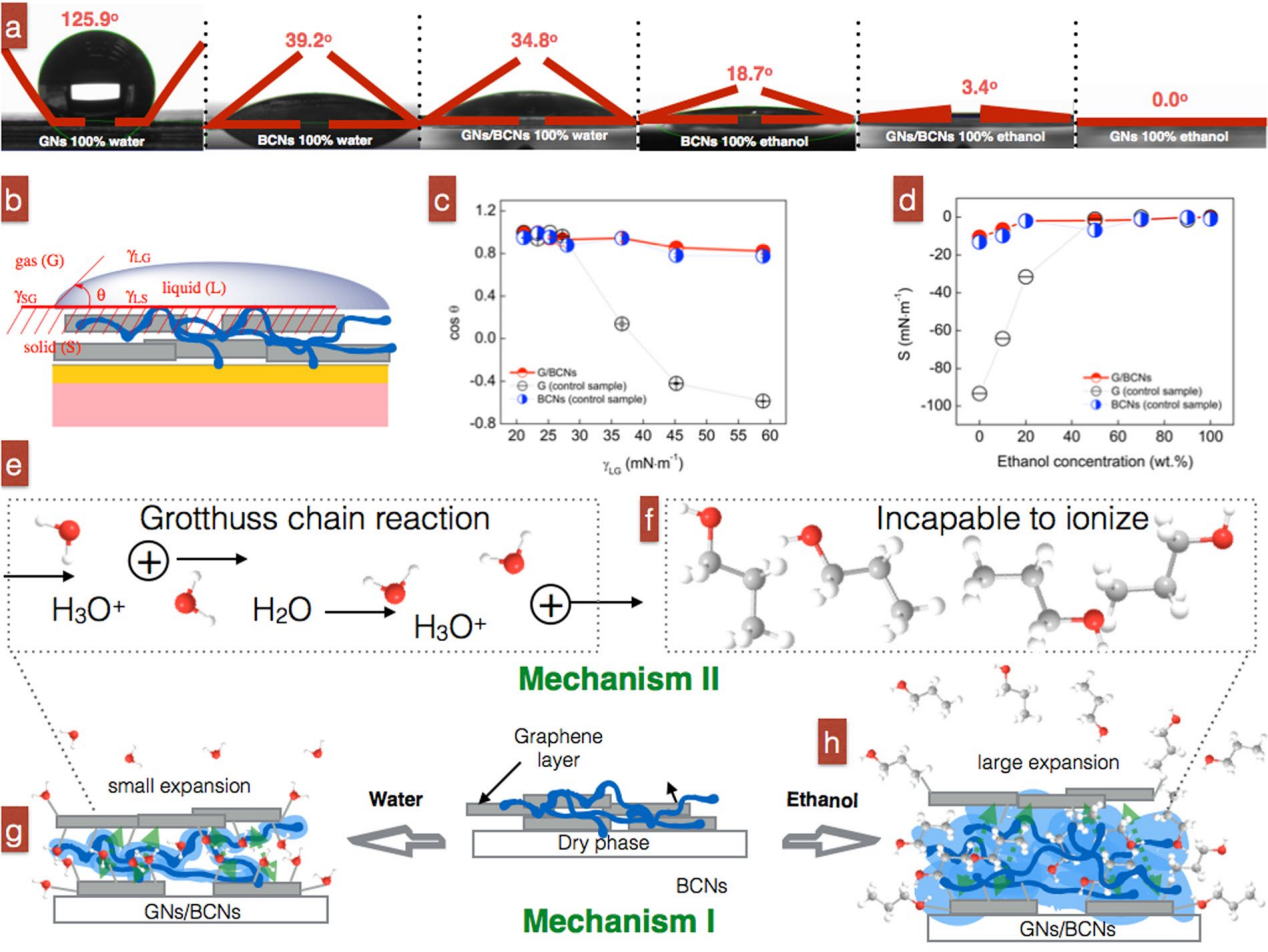
(**a**) Contact angles between pure ethanol and pure water for GN, GN/BCN and BCN films. (**b**) Illustration of parameters in contact angle measurements. (**c**) Dependence of cos θ as a function of surface tension γ_LG_. (**d**) Spreading coefficient of various liquids on solid samples (GN/BCN, GN and BCN) with respect to ethanol concentration. (**e**,**f**) Mechanism II explaining ionization of liquids. (**g**–**h**) Mechanism I explaining resistance changes induced by wetting of BCNs in GN/BCN sensor devices.

Electrical resistance change of GN/BCN sensors is thought to relate to mass diffusivity in the porous GN/BCN film. Surface pores of GN/BCN thin films permits the penetration of liquid into the internal microstructure. The change in resistance is related to the intrinsic (volume, dielectric) properties of target liquids. Both effective diffusivity D_e_ (cm^2^·s^−1^) and spreading coefficient *S* (mN·m^−1^) can be used to evaluate diffusivity:

5 $${D}_{e}=\frac{D{\varepsilon }_{t}\delta }{\tau }$$

6 $$S={\gamma }_{SG}-{\gamma }_{SL}-{\gamma }_{LG}={\gamma }_{LG}(\cos \,\theta -1),$$

where D is the diffusion coefficient of liquid filling the pores, ε_t_ is the porosity, δ is the constrictivity (dimensionless), τ is the tortuosity (dimensionless) and γ refers to the surface tension. SG, SL, LG represent the interface between each of the two phases: solid (S), gas (G) and liquid (L) (Fig. 6b). Here, the spreading coefficient *S* determines the spontaneous spreading for a drop of liquid placed on a solid substrate.

We measured the changes in resistance when we inserted a 225-nm-thick GN/BCN sensor into three most common types of alcohol used in the laboratory to check if it depended on the type of alcohol (Fig. S1). The initial resistance was recorded for around 20 s. Then, the sensor was inserted into the target liquid for 3 s, followed by removing the sensor and drying in air (around 60% relative humidity (RH%)). The same sensor was used throughout all experiments. Prior to each type of measurement, the sensor was dried completely by compressed air with 20 RH%. We found there that the GN/BCN sensor could detect different alcohols. For example, resistance change was the highest for methanol, followed by ethanol and isopropanol. We ascribe the similarity to the similar physical properties as shown in Equations 5 and 6. However, we are unable to identify from where the difference originated. An in-depth study of computation of the multi-physical mechanics is necessary.

Figure 6a shows representative contact angles of pure ethanol and pure water sitting on GN, GN/BCN or BCN films. Overall, GN/BCN films had higher wettability (small contact angle) to ethanol than to water (3.4° < 34.8°). Note that rapid absorption of ethanol into the film (coupled with fast evaporation of ethanol) can make the angle measurement difficult and inaccurate. Therefore, θ values only present the initial value at the moment just after the droplet contacted the film surface. Differences in wettability result in differences in penetration and interlayer expansion of the liquid in films. Therefore, wettability likely contributes considerably to the performance of the sensing device.

We also correlated the contact angle and surface tensions of liquid ethanol/water mixtures on the surface of GN/BCN, GN and BCN films (Fig. 6c). For all the films, the cos θ on GN/BCN films decreases with increasing surface tension γ_LG_, suggesting that ethanol content (resulted in different surface tensions) was responsible for the changed wettability (Fig. 6d). When exposed to various concentrations of liquid ethanol, GN/BCN films had a high cos θ, indicating a high absorbance to liquids and subsequently a large volume of liquids in the devices. Figure 6d shows that *S* increases as ethanol content increases; for example, GN/BCN films had the highest *S* in pure ethanol, suggesting that GN/BCN films had the highest ethanol absorbance, which agrees well with our analysis of contact angle/surface tension measurements.

We confirmed that disparity in spreading coefficient results in a difference in volume of absorbed liquids. BC films have previously been reported to have a high swelling expansion of up to 6225% from its dried state due to a hydrophilic functional surface^38^. Humidity sensors based on hydrophilic polyvinyl alcohol/carbon nanotube composites have also been reported to have a swelling expansion effect due to the hydrophilic polymer compound in devices^39^. Likely the reason that our GN/BCN films absorbed different volumes of liquids is because of a disparity in wettability upon contact with target liquids or vapors (Fig. 6g,h). It is their large number of hydrophilic groups, including carboxyl groups, hydroxyl groups and air vacancies, in the network of BCN fibers that yields their high absorbance to both ethanol and water^40^.

Secondly, it is believed that ionization of liquids play a vital role for conductance of GN/BCN films (Mechanism II). It is well known that electrical conductance in graphene or graphene-oxide increases if water is absorbed onto graphene. This is why it is often used as a material aimed for humidity sensors. The mechanism behind the resistance change is called water-induced ionic conductivity. Ionization of water creates hydronium ions (H_3_O^+^) that behave as charge carriers^41^:

Reac 1 $$2{{\rm{H}}}_{2}{\rm{O}}\to {{\rm{H}}}_{3}{{\rm{O}}}^{+}+{{\rm{OH}}}^{-}$$

These carriers are mobile, making the electrical path more conductive via a Grotthuss chain reaction through proton transfer (Fig. 6e):

Reac 2 $${{\rm{H}}}_{2}{\rm{O}}+{{\rm{H}}}_{3}{{\rm{O}}}^{+}\to {{\rm{H}}}_{3}{{\rm{O}}}^{+}+{{\rm{H}}}_{2}{\rm{O}}$$

This also generates its high dielectric constant up to 80.4 (unit-less), which means that substances whose molecules contain ionic bonds will tend to dissociate, yielding solutions containing ions. When Freeman *et al*. measured the generation of free ions of different solvents induced by radiation, they correlated the dielectric constant with the yield of free ions to confirm that the relationship is proportional. We can compare the values for ethanol and water in this report^42^.

Under this circumstance, because GN/BCN films become more conductive (low resistance) if water is absorbed, the large number of existing -COONa groups in BCNs could aid with proton migration. The increase in conductance (Grotthuss chain reaction) will cancel out the increase in resistance caused by Mechanism I, making the change in resistance (sensitivity) of GN/BCN films relatively low. This explains why GN/BCN films have a low sensitivity to water.

In contrast, when GN/BCN films absorbed ethanol, they did not become more conductive (dielectric constant 24.3) (Fig. 6f). This is because ethanol cannot be ionized, and therefore, the number of charge carriers in the GN/BCN network does not increase Instead, the absorbed ethanol produces more “insulating” segments in the conductive network, resulting in a GN/BCN sensor with high sensitivity.

## Conclusion

We successfully built flexible, transparent, highly sensitive GN/BCN thin film sensor devices with excellent alcohol recognition performance. Electrical tests under different liquid environments showed that the GN/BCN sensor exhibited ultrahigh sensitivity of up to 12400% in response to pure ethanol in a vapor phase compared to a 920% sensitivity response to pure water. We ascribed the altered wettability of BCN films and the ionization of liquids as the reasons for their excellent sensing performance.

## Materials and Methods

Non-polar, hydrophobic GN powder (N002-PDR, Angstronmaterials Company) was used as received. 5 wt.% sodium hypochlorite (NaClO) solution and sodium bromide (NaBr) powders were purchased from RICCA Chemical Company. 2,2,6,6-tetramethyl-1-piperidinyloxy (TEMPO, 98% purity) was purchased from Sigma-Aldrich Company, and ethanol (100 vol.%) was purchased from VWR International. Water was purified by distillation in a Milli-Q (Advantage A10 model) system. BC cubic gels were produced by Thai Agri Foods Public Company Limited. The cubes were cleaned by soaking in distilled water for 15 d; the water was changed every 24 h. Cleaned gels had a BC concentration of 0.5 wt.%. BCNs were extracted from BC hydrogels using a facile TEMPO-mediated blending process. For this, 58.15 g of bacterial cellulose hydrogels, 72.5 g of water and 100 g of ice were blended together by a commercial blender for 5 min. Then, 0.1072 g of TEMPO, 0.7572 g of NaBr and 40.956 g of NaClO solution were mixed with the slurry and stirred at 500 rpm for 20 min. The TEMPO-mediated suspension was sonicated (500 W, 20 kHz, Cole-Parmer Company) at 765 W for 1 min. Next, the TEMPO/NaClO/NaBr/BCNs aqueous colloidal solution was centrifuged (Centrifuge 5810, Eppendorf Company) at 10,000 rpm for 10 min. Liquid chemicals were removed by repeated centrifugation and the obtained wet treated BCN slurry was dialyzed against pure water for 20 d. The concentration of BCNs was adjusted to 0.45 wt.% by adding water. GN/BCN colloidal solutions were prepared by mixing GN powder and BCN suspension at various concentrations of GN for 20 min using an ultrasonicator. GN/BCN suspensions were centrifuged at 1000 rpm for only ~10 s to remove large aggregates and the remaining homogenous GN/BCN colloidal solutions were used to assemble the sensors.

Sensor devices were assembled by vacuum-filtration of GN/BCN colloidal solutions. Filtration with a 47-mm Wheaton filtration assembly and polycarbonate filter substrate took 1 min, leaving a thin wet GN/BCN film supported by a membrane filter. Afterwards, a wet GN/BCN film supported by a PC membrane was placed on top of a plastic substrate (0.005, Clear Dura-Lar Brand) with the GN/BCN film facing the plastic substrate. This substrate was previously sputtered with a titanium/gold (10, 100 nm thick) interdigitated electrode with a metal-sputter system (Equipment Support Co., Cambridge, England). Next, samples and several layers of soft tissue paper were laminated between two metal cells (2400 g) and dried in a vacuum oven at 60 °C for 4 h. Finally, the supporting materials, including the tissue paper and membrane filter, were peeled away from the GN/BCN layer.

TEM images were taken using a Tecnai Twin microscope (FEI). Fourier transform-infrared (FT-IR) spectroscopy measurements were performed on a Nicolet iS10 (Thermoscientific Inc). Ultraviolet-visible (UV-vis) spectroscopy measurements were measured by a Cary100 ConC UV-vis spectrophotometer (Agilent Technologies). Thicknesses of GN/BCN films were measured by a DEKTAK*8 profilometer (Veeco Company) equipped with a 12.5-μm radius tip. Sheet resistances were measured using a CMT-SR2000N four-probe system purchased from Materials Development Corporation. Data were averaged based on ten measurements at different locations. Surface tensions were averaged based on twenty times of measurements by dynamic surface-tension-ring method on a Kruss K100 tensiometer (Kruss Company) operating at 20 °C. Contact angles were measured by DSA100 equipment purchased from Kruss Company. The sensing performance of GN/BCN sensors was evaluated using a homemade Climatic Test Chamber (30, 45, 60 cm^3^) operating at 21.5 °C equipped with an air humidifier (LB88 dual, Beurer Company). Electrical resistance of the sensors was measured using a U1281A True RMS Multimeter (Keysight Company).

## Electronic supplementary material

Supporting information
