# Supplementary material for: Alcohol Recognition by Flexible, Transparent and Highly Sensitive Graphene-Based Thin-Film Sensors
Source: Sci Rep. 2017 Jun 28;7:4317. doi: 10.1038/s41598-017-04636-2 (PMC5489480; doi:10.1038/s41598-017-04636-2)
Supplement: Supplementary file 1 — Supporting information [file 41598_2017_4636_MOESM1_ESM.doc]

**Supporting Electronic Information:**

**Alcohol Recognition by Flexible, Transparent and Highly Sensitive Graphene-Based Thin-Film Sensors**

*Xuezhu Xu,^a^ Jian Zhou,^a^ Yangyang Xin,^a^ Gilles Lubineau,^a,*^ Qian Ma, ^b,c^ Long Jiang,^b^*

*^a^King Abdullah University of Science and Technology (KAUST), Physical Science and Engineering Division, COHMAS Laboratory, Thuwal 23955-6900, Saudi Arabia*

*^b^North Dakota State University, Department of Mechanical Engineering, Fargo, ND 58102, United States*

*^c^College of Textile and Clothing, Yancheng Institute of Industry Technology, Yancheng 224005, P. R. China*

**Corresponding author: Prof. G. Lubineau (*[*gilles.lubineau@kaust.edu.sa*](mailto:gilles.lubineau@kaust.edu.sa)*).*


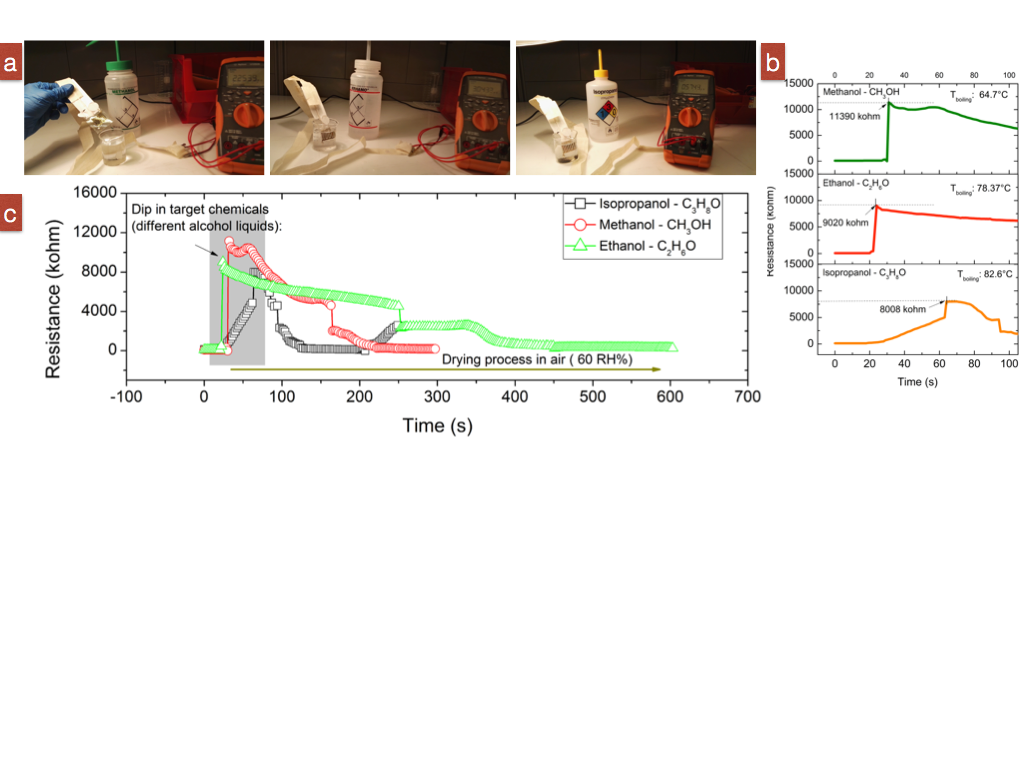
Fig. S1. Selectivity to different alcohols by a 225-nm-thick GN/BCN sensor. (a) methanol, alcohol and isopropanol (from left to right); (b) initial stage and (c) whole process for a piece of GN/BCN sensor before and after putting in target liquids.
